# Real-World Neoadjuvant Systemic Therapy Utilization and Treatment Patterns in Patients with Early-Stage or Locally Advanced Triple-Negative Breast Cancer in Greece—The TRINITY Study

**DOI:** 10.3390/cancers17244023

**Published:** 2025-12-17

**Authors:** Konstantinos Papazisis, Christos Christodoulou, Flora Zagouri, Ippokratis Korantzis, Ioannis Boukovinas, Anna Koumarianou, Angelos Koutras, Eleni Timotheadou, Giannis Mountzios, Loukas Kontovinis, Ioannis Binas, Alkistis Papatheodoridi, Eleni Zairi, Ilias Gountas, Danai Ktena, Charalampos Athanasopoulos, Athanasios Kotsakis, Emmanouil Saloustros

**Affiliations:** 1 Euromedica General Clinic, 54636 Thessaloniki, Greece; konstantinos.papazisis@gmail.com (K.P.); l.kontovinis@oncomedicare.com (L.K.); 2 Metropolitan Hospital, 18547 Athens, Greece; c_christodoulou@yahoo.gr (C.C.); ioannisbinas@gmail.com (I.B.); 3 Department of Clinical Therapeutics, “Alexandra” General Hospital of Athens, School of Medicine, National Kapodistrian University of Athens, 11528 Athens, Greece; fzagouri@med.uoa.gr (F.Z.); alkipapath@med.uoa.gr (A.P.); 4 Medical Oncology Department, St Luke’s Hospital, 55236 Thessaloniki, Greece; korangr@yahoo.com (I.K.); zairi.eleni@gmail.com (E.Z.); 5 Department of Medical Oncology, Bioclinic Hospital, 54639 Thessaloniki, Greece; ibouk@otenet.gr; 6 Hematology-Oncology Unit, Fourth Department of Internal Medicine, “Attikon” University General Hospital, Medical School, National and Kapodistrian University of Athens, 12462 Athens, Greece; akoumari@yahoo.com; 7 Division of Oncology, Department of Medicine, School of Medicine, General University Hospital of Patras, 26504 Rion, Greece; angkoutr@otenet.gr; 8 Department of Medical Oncology, “Papageorgiou” General Hospital, Aristotle University of Thessaloniki, 54124 Thessaloniki, Greece; timotheadou@auth.gr; 9 Fourth Department of Medical Oncology and Clinical Trials Unit, Henry Dunant Hospital Center, 11526 Athens, Greece; gmountzios@gmail.com; 10 MSD, External Affairs, 17456 Athens, Greece; ilias.gountas@merck.com (I.G.); danai.ktena@merck.com (D.K.); 11 MSD, Medical Affairs, 17456 Athens, Greece; 12 Department of Oncology, University General Hospital of Larissa, 41334 Larissa, Greece; thankotsakis@hotmail.com (A.K.); esaloustros@yahoo.gr (E.S.)

**Keywords:** triple-negative breast cancer, early stage, locally advanced, neoadjuvant systemic therapy, real-world evidence, pathological complete response

## Abstract

Background: Guidelines recommend neoadjuvant systemic therapy (NST) as the preferred treatment approach for stage II–III triple-negative breast cancer (TNBC), an aggressive form of breast cancer (BC) that lacks specific therapeutic targets. This study primarily aimed to assess the NST adoption among stage II–III TNBC patients in Greece under real-world conditions during the pre-immunotherapy era. Methods: This multicenter, observational, retrospective chart review included 230 female patients (≥18 years) with early-stage or locally advanced TNBC across 10 public and private BC reference centers over 6.5 years. Data included demographics and clinical characteristics at diagnosis, treatment details, clinical outcomes, and survival status. Descriptive statistics followed by uni/multivariate analyses were performed. Survival outcomes were assessed using survival analysis methods. Results: Women with stage II (67.4%) or stage III (32.6%) TNBC were included, with a median age of 53.1 years (range 23.9–84.1). Patients received NST [113 (49.1%)] and non-NST [117 (50.9%)]. NST utilization was significantly associated with larger tumor size and *BRCA1/2* testing and status. Overall, 43.9% underwent *BRCA1/2* testing, and 32.7% of those were positive for a *BRCA1/2* mutation. More than half of the patients (*n* = 61) achieved pathological complete response (pCR) following NST. Event rates were lower with NST (16.8%) versus without (24.8%). Utilization increased over time, peaking at 63.5% in 2020–2022. Conclusions: NST use showed moderate uptake with notable practice variations, emphasizing the need for multidisciplinary strategies to improve guideline adherence. Over half achieved pCR post-NST, setting a benchmark for TNBC care. Ongoing real-world monitoring is vital to guide long-term outcomes.

## Simple Summary

Current treatment guidelines endorse neoadjuvant systemic therapy (NST) for stage II–III triple-negative breast cancer (TNBC), an aggressive form of breast cancer. The primary aim of this study was to assess the level of NST use in routine clinical practice in Greece for patients with stage II–III TNBC, before newer targeted therapies, such as immunotherapies, became available. By reviewing the medical records of 230 patients diagnosed between 2016 and 2022 at ten hospitals across Greece, we found that approximately half of the patients received NST, with significant variations among different centers. Our findings highlight the need for strategies to improve adherence and promote the consistent application of treatment guidelines across all healthcare settings, ensuring equal access to optimal care for all patients, especially as new treatments for early-stage or locally advanced TNBC continue to emerge.

## 1. Introduction

### Background

Breast cancer (BC) is the most commonly diagnosed cancer among women worldwide, with approximately 375,000 new cases (29.4% of all cancer cases) and over 95,000 deaths (16.7 of all cancer mortality) annually across Europe. In Greece, an estimated 8987 new patients were diagnosed with BC in 2022, making it the most prevalent cancer, while 2431 deaths in the same year ranked it as the third leading cause of cancer mortality [1,2].

Triple-negative breast cancer (TNBC), accounting for approximately 15–20% of all BC cases, represents a heterogeneous subgroup of BC defined by the absence of the expression of *estrogen receptor (ER)* and progesterone receptor (PR), as well as of *human epidermal growth factor receptor 2 (HER2)* overexpression. Despite its lower incidence compared with other BC subtypes, TNBC is characterized by a distinctly aggressive biological and clinical behavior, translating into higher and earlier rates of local recurrence and visceral metastasis, and shorter overall survival in the metastatic setting [3,4,5,6].

Given these challenges and the limited treatment options due to the absence of specific molecular actionable targets, therapeutic decision-making represents a crucial point in the early stages of TNBC [4]. Treatment of early-stage or locally advanced (stages II–III) TNBC traditionally involved surgical resection followed by adjuvant chemotherapy and/or radiotherapy, while in the modern era, neoadjuvant chemotherapy is increasingly becoming a standard treatment approach [7,8,9,10,11].

According to the NCCN Breast Cancer Guidelines (2024) [8], patients with TNBC, if at clinical tumor stage ≥ cT2 or clinical nodal stage ≥ cN1, may receive preoperative systemic therapy. This approach facilitates breast conservation, allowing for operability in advanced tumors, and provides prognostic insight, particularly in TNBC. Patients with residual disease are considered for additional adjuvant regimens. Recommended preoperative and adjuvant treatments for operable or high-risk TNBC include dose-dense doxorubicin/cyclophosphamide followed by paclitaxel, docetaxel/cyclophosphamide, or olaparib for *BRCA1/2* mutations. High-risk stage II–III cases may benefit from pembrolizumab-based combinations, with capecitabin or platinum-taxane regimens for residual or selected preoperative cases [9,10,11,12]. The ASCO guideline (2022) [13] recommends anthracycline and taxane-based chemotherapy regimens as well as pembrolizumab, an immune checkpoint inhibitor, for neoadjuvant chemotherapy in high-risk early-stage TNBC (stage II/III).

However, increasing efforts to expand the currently limited therapeutic landscape have led to the introduction of platinum compounds and immunomodulatory agents into the neoadjuvant setting, rapidly reshaping the treatment algorithms of the disease. Notably, the positive results of the pivotal Phase III KEYNOTE-522 trial have significantly altered the treatment paradigm for stage II and III TNBC by establishing that the addition of pembrolizumab to neoadjuvant chemotherapy as a new standard of care significantly improves event-free survival (EFS) for stage II–III TNBC [14,15].

Despite the recent advances in the neoadjuvant treatment landscape for stage II–III TNBC, it presents treatment challenges due to several factors, such as a lack of targeted therapeutic options, monitoring tumor response, preoperative routine checkup, etc., which may still hinder the adoption of neoadjuvant systemic treatment (NST) in routine clinical practice [16]. However, limited data exist on the real-world utilization of NST and treatment patterns in pre- and post-surgery settings for managing early-stage or locally advanced TNBC worldwide [16,17,18,19,20,21,22,23,24].

The development of bioelectronic sensing methods has enabled precise, real-time assessment of BC response. The label-free Metas-Chip provides high-accuracy detection of micrometastases, offering a non-conventional route to identify minimal residual disease. This technology is vital for improving pathological complete response (pCR) prediction and guiding individualized therapy in neoadjuvant TNBC management [25].

In Greece, BC care is delivered by various healthcare providers, including public and private hospitals, potentially resulting in variations in treatment practices. Given the lack of evidence on NST utilization and the extent of adoption of contemporary international guidelines for treating stage II–III TNBC, there is an urgent need to explore these practices. Understanding and defining best practices is essential to ensure consistent and optimal care for TNBC patients across different healthcare settings.

This study offers novel insights by systematically evaluating the real-world utilization of NST and adjuvant systemic treatment (AST) specifically for early-stage and locally advanced TNBC across multiple clinical institutions in Greece. It documents detailed treatment patterns in both pre- and post-surgical settings, highlighting variations in clinical practice that have not been previously documented nationally. Additionally, it sought to provide a comprehensive view of the local NST utilization landscape in the pre-immunotherapy era.

This study aimed to assess the utilization of NST and/or adjuvant systemic treatment (AST) to treat early-stage or locally advanced (stages II–III) TNBC in routine clinical practice, document the treatment patterns in pre- and post-surgery settings, as well as to identify any variations in treatment approaches between different institutions across Greece. Additionally, it sought to provide a comprehensive view of the local NST utilization landscape in the pre-immunotherapy era.

## 2. Materials and Methods

### 2.1. Study Design

TRINITY was a multicenter, observational, retrospective chart review study involving female patients diagnosed with early-stage or locally advanced TNBC over approximately a 6.5-year period, prior to the first approval of an immunotherapy-based combination in the neoadjuvant setting on 19 May 2022. The study was conducted at 10 public and private BC reference centers from various regions across Greece, selected to reflect clinical practice variations and to provide a representative sample of the Greek population. Patients’ medical records were reviewed for eligibility by the investigators following consecutive sampling in reverse chronological order, based on the date of TNBC diagnosis, starting with the most recently diagnosed patients on 19 May 2022, and moving back to 4 January 2016. Eligible patients attending routine clinical visits at participating sites during the selection period were also considered for study inclusion. The retrospective observation period for each participating patient extended from the date of informed consent obtainment (ICF) (for patients alive at the time of chart abstraction onset) or death (for patients deceased at the time of chart abstraction onset) back to the date of early-stage or locally advanced TNBC diagnosis.

### 2.2. Study Population

Eligible for participation in the study were females aged ≥ 18 years diagnosed with stage II or III TNBC between 5 January 2016, and 19 May 2022 (the date of pembrolizumab’s approval by EMA in early-stage TNBC), who had undergone definitive BC surgery and had at least 6 months of follow-up data post-surgery. Patients receiving treatment for early-stage or locally advanced TNBC within the context of a clinical trial or receiving off-label immunotherapy or targeted therapy were excluded.

### 2.3. Data Collection

All data pertaining to the objectives of the study were abstracted from patients’ medical records and mainly included patient demographics and clinical characteristics at the time of diagnosis, treatment details (NST and/or AST utilization, surgery type, radiation therapy, etc.), clinical outcomes (pathological complete response [pCR] at the time of definitive surgery, disease progression, local or distant recurrence post-surgery) and survival status. Data recorded at TNBC diagnosis served as baseline data. For variables lacking data on the date of TNBC diagnosis, the data closest to the date of TNBC diagnosis and prior to or on the date of treatment onset for TNBC management (i.e., NST onset or definitive surgery, as applicable) served as baseline data. Data were entered by trained personnel at the sites into electronic case report forms (eCRFs) and stored in a study-specific, password-protected, web-based electronic data capture (EDC) system.

### 2.4. Study Objectives

The primary objective of the study was to explore the utilization of NST and/or AST among patients with stage II–III TNBC and to describe commonly used treatment regimens, types of surgery (mastectomy or BCS), radiation therapy use, and the demographic and clinical profile of patients with stage II–III TNBC. Secondary objectives included investigating variations in NST use among institutions and regions across Greece and pathological complete response (pCR) rates at the time of definitive surgery post-NST. In addition, time-to-event outcomes—defined as the time from definitive surgery to the first occurrence of (local or distant) recurrence, disease progression, or death from any cause—were evaluated. Associations between various baseline demographic and clinical characteristics and the above outcomes were also explored.

### 2.5. Statistical Analysis

Given the observational nature of the study, no formal hypothesis testing was performed. The sample size calculation was based on precision estimates for the primary outcomes, i.e., the frequencies of treatment modalities, demographic, and clinical characteristics. Assuming a worst-case scenario of estimating a percentage of 50%, a sample size of 230 patients provided a maximum margin of error of approximately 6.5%, representing a scientifically acceptable level of precision.

Summary statistics were used for all descriptive variables. Categorical variables were summarized as relative and absolute frequencies, while continuous variables were summarized based on the number of available observations (*n*), mean, standard deviation (SD), median, and range (minimum, maximum). Associations between patient characteristics at the time of diagnosis and NST use or pCR were assessed using univariable and multivariable logistic regression models. Multivariable analysis employed a stepwise procedure, including covariates with a *p*-value < 0.1 in univariable analysis or considered clinically significant. Variables with a missing rate > 20% were also excluded from the initial step of the stepwise procedure. In addition, variables with an unbalanced distribution (<10% of the total observations in any of the defined variable levels) were also excluded from the multivariable analyses. Odds ratios (ORs) and their corresponding 95% confidence intervals (CIs), along with *p*-values, were estimated for each variable, with *p* < 0.05 considered statistically significant. Time-to-event outcomes were estimated using the Kaplan–Meier method and are presented as median and 95% CIs. Comparisons were performed among NST-treated patients according to pCR status using the log-rank test. All statistical analyses were performed using SAS^®^ version 9.4.

## 3. Results

### 3.1. Patient Demographic and Clinical Characteristics

A total of 230 women diagnosed with stage II to III TNBC between 1 January 2016, and 19 May 2022, across 10 private and public BC reference centers in Greece, were enrolled in the study between 7 October 2022, and 20 July 2023. Table 1 summarizes the main demographic and clinical characteristics of the study population at the time of diagnosis, both overall and by NST utilization.

The median age at diagnosis was 53.1 years (range 23.9–84.1). The majority of patients were postmenopausal (55.7%) and had an Eastern Cooperative Oncology Group performance status (ECOG PS) of 0 (88.7%). Additionally, 33.0% of patients reported a family history of BC. Only 23.5% were classified as obese (body mass index [BMI] ≥ 30 kg/m^2^).

Overall, 67.4% of patients were diagnosed at stage II, while 32.6% were diagnosed at stage III. Patients had a median of 1 tumor (range 1.0–8.0) with a median tumor size of 27.5 mm. Histologically, the majority of tumors were Grade 3 (80.0%), with 16.5% being Grade 2. More than half of the patients (58.3%) had lymph node involvement.

Testing for *BRCA1/2* mutations was conducted in 43.9% of patients. Among those tested, 32.7% were positive for mutations; 31.7% for *BRCA1* and 5.9% for *BRCA2* mutations.

Notably, patients at public centers were relatively older, with a median age at diagnosis of 58.0 years compared to 48.0 years at private centers. A higher percentage of patients at private centers were diagnosed at stage II (79.55%), whereas more patients at public centers were diagnosed at stage III (48.98%). Lymph node involvement was higher among patients treated in public centers (68.37% vs. 50.76%). Finally, *BRCA* testing at the time of diagnosis was more common in private centers (55.3% vs. 28.57%).

### 3.2. Surgery and Radiation Therapy

Mastectomy was the most common surgical procedure, performed in 67.0% of patients (76.53% in public and 59.85% in private centers), while the rest underwent BCS (i.e., lumpectomy; Table 1), with no apparent difference between those treated with NST and those who were not. Among those who underwent a mastectomy, 74.7% had a unilateral mastectomy and 25.3% had a bilateral mastectomy. Interestingly, a slight, gradual yet consistent shift towards less invasive surgical options was observed from 2016 to 2022. In 2016–2017, 70.6% of patients underwent mastectomy, while 29.4% opted for BCS. By 2018–2019, the percentage of mastectomies decreased to 67.0% and BCS increased to 33.0%. This trend continued in 2020–2022, with 63.5% undergoing mastectomy and 36.5% BCS.

As expected, no radiation therapy was documented pre-operatively, whereas 58.3% of all patients received radiation therapy post-surgery, with a median of 1 session (range 1.0–2.0).

### 3.3. Systemic Treatment Patterns and Characteristics

**Figure 1:**
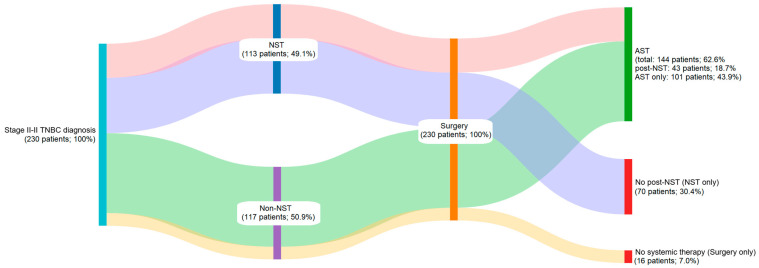
Sankey diagram of treatment patterns (*N* = 230). AST, adjuvant systemic therapy; NST, neoadjuvant systemic therapy; TNBC, triple-negative breast cancer.

**Figure 2:**
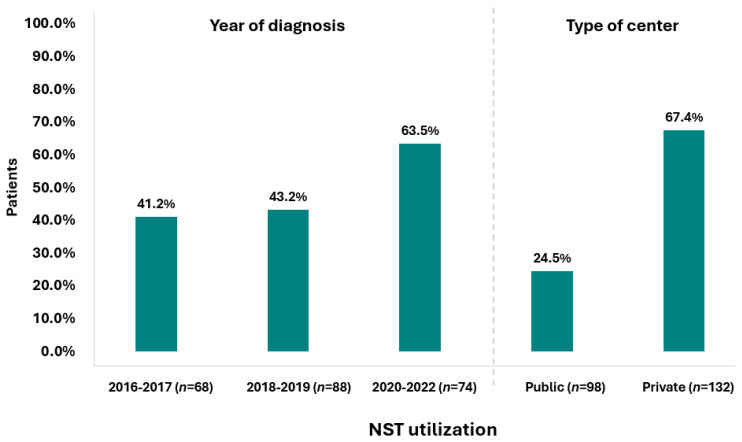
NST utilization by year of diagnosis and type of center (*N* = 230).

A Sankey diagram was constructed to depict real-world treatment among patients with stage II–III TNBC. The diagram represents the distribution and transitions of patients (*N* = 230) across key therapeutic stages, with node height and connecting band width proportional to the number of patients at each stage. Patients were divided into those receiving NST and those managed without NST, followed by surgery and subsequent receipt or omission of AST. Overall, 49.1% (95% CI: 42.5–55.8%) of the study population received NST (median duration: 105.0 days [range 0–176 days]; median of 8.0 cycles [range 1.0–16.0 cycles]), either as a standalone treatment or followed by AST. Specifically, 30.4% (95% CI: 24.6–36.8%) underwent only NST, while 18.7% (95% CI: 13.9–24.3%) also received AST after surgery (post-NST setting). Conversely, 50.9% of patients underwent upfront surgery without any prior NST. Of these, 43.9% (95% CI: 37.4–50.6%) subsequently received AST, while 7.0% (95% CI: 4.0–11.1%) did not receive any systemic treatment post-surgery. Notably, 62.6% of patients received AST (median duration: 119.0 days [range 0.0–513.0 days]; median of 8 cycles [range 1.0–26.0]), either alone or following NST (post-NST setting) (Figure 1). Data indicate a rising trend in NST use over the years, with the highest utilization observed during the 2020–2022 period, reaching 63.5% (Figure 2). Notably, NST utilization also varied by center from 0.0 to 93.8%.

When comparing their demographic and clinical characteristics at diagnosis, patients who received NST were generally younger with a median age at diagnosis of 48.0 years compared to 58.5 years for those who did not receive NST, and more often premenopausal (54.0% vs. 25.6%). Additionally, these patients exhibited a higher prevalence of family history of BC (39.8% vs. 26.5%), more frequently presented with Grade 3 tumors (82.3% vs. 77.8%), and had larger tumors at diagnosis (median size: 30.0 mm vs. 25.0 mm). They also showed elevated Ki 67 indices (median: 70.0% vs. 50.0%) and were more commonly diagnosed at Stage II (70.8% vs. 64.1%). Moreover, a substantially higher proportion of NST-treated patients had undergone *BRCA* testing (63.7% vs. 24.8%), with a higher incidence of positive *BRCA1* and/or *BRCA2* mutations (36.1% vs. 24.1%) (Table 1).

Univariable analysis of associations between patient characteristics at the time of diagnosis and NST use is presented in Table S1. Subsequent stepwise multivariable analysis revealed that larger tumor size (OR [95% CI]: 1.03 [1.01–1.06]; *p* = 0.008), being tested for *BRCA* mutations at diagnosis (OR [95% CI]: 3.28 (1.62–6.64), *p* < 0.001) and being treated at a private institution (OR [95% CI]: 5.43 [2.85–10.36]; *p* < 0.001) were independently associated with increased odds of NST use. Conversely, age at diagnosis was not significantly associated with NST use (OR [95% CI]: 0.98 [0.96–1.01]; *p* = 0.128) (Table S2).

Table 2 shows the treatment patterns for patients with early-stage or locally advanced TNBC in the NST and AST settings. In the NST, the most commonly used combination was anthracyclines, cyclophosphamide, platinum compounds, and taxanes (59.3%), followed by anthracyclines, cyclophosphamide, and taxanes (32.7%). In the AST setting, among patients with available data on treatment (*N* = 126), the combination of anthracyclines, cyclophosphamide, and taxanes (31.0%) and pyrimidine analogues (i.e., capecitabine; 31.0%) were equally used, followed by anthracyclines, cyclophosphamide, platinum compounds, and taxanes used in 12.7% of the patients.

In more detail, one-third of patients (34.5%) received epirubicin, cyclophosphamide, paclitaxel, and carboplatin in the neoadjuvant setting. Epirubicin, cyclophosphamide, docetaxel, and carboplatin were the second most common treatment combination received (by 21.2% of patients), while 16.8% received epirubicin, cyclophosphamide, and docetaxel. Post-NST, 88.4% of those receiving additional AST were prescribed capecitabine. In patients who received only AST postoperatively without prior NST, the predominant chemotherapy regimen was epirubicin, cyclophosphamide, and docetaxel, used in 32.5% of the cases. Additional AST regimens included epirubicin, cyclophosphamide, docetaxel, and carboplatin for 13.3% of the patients, and epirubicin, cyclophosphamide, and paclitaxel for 8.4% of the patients (Table 2).

### 3.4. PCR

Following NST treatment (*N* = 113), 54.0% of patients achieved a pCR by the time of definitive surgery. Baseline and clinical characteristics of NST-treated patients overall and by pCR are presented in Table S3.

When stratified by lymph node involvement and clinical stage, the pCR rates showed no apparent differences. Specifically, patients without lymph node involvement had a pCR rate of 52.3%, while those with lymph node involvement had a pCR rate of 55.1%. Similarly, the pCR rate was 55.0% in stage II and 51.5% in stage III patients. Consistently, univariable analysis identified no significant associations between lymph node involvement, clinical stage, and other baseline demographic and/or clinical characteristics and achieving pCR among NST-treated patients, except for *BRCA testing* and *BRCA1/BRCA2* status (Table S4). Testing positive for *BRCA1* mutations, as well as testing positive for *BRCA1* and/or *BRCA2* mutations at diagnosis, was associated with higher odds of achieving pCR (OR [95% CI]: 6.30 [2.00–19.86]; *p* = 0.0017 and (OR [95% CI]: 6.77 [2.15–21.29]; *p* = 0.0011, respectively). However, these estimates should be interpreted with caution, as the wide confidence intervals indicate limited precision due to the imbalance in subgroup sizes.

In relation to the most utilized NST treatment regimens, pCR rate was 59.7% (40/67) for patients previously treated with anthracyclines, cyclophosphamide, platinum compounds, and taxanes, and 40.5% (15/37) for patients previously treated with anthracyclines, cyclophosphamide, and taxanes.

Finally, among those with pCR (*N* = 61), 41.0% subsequently received adjuvant radiotherapy, 39.3% did not receive subsequent treatment, and only 9.8% received either AST or AST and adjuvant radiotherapy. Conversely, among those with residual disease at the time of definitive surgery (*n* = 48), 54.2% received both AST and radiotherapy, and 31.3% received adjuvant radiotherapy; 8.3% received AST, and 6.25% did not receive any adjuvant therapy.

### 3.5. Time to Recurrence, Disease Progression, or Death Post-Surgery

At a median follow-up of 39.3 months (range < 0.1–85.9) from definitive surgery, 20.9% of the overall study population (48/230) experienced (local or distant) recurrence, disease progression, or death from any cause. The median time to event was not reached (NR), and the 5-year rate of remaining free from such events was 74.5% (95% CI: 67.0–80.6%).

**Figure 3:**
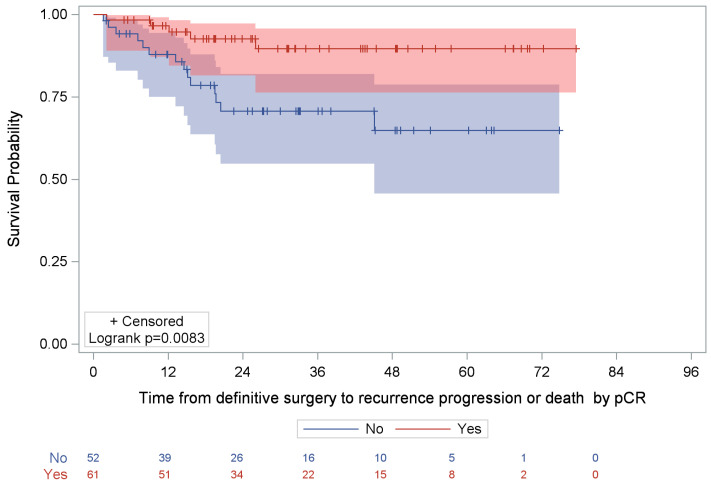
Kaplan–Meier analysis of time to recurrence/disease progression or death post-surgery by pCR status among NST-treated patients. NR, not reached; pCR, pathological complete response. **Note:** Shaded areas (red and blue) represent the 95% confidence interval of the respective Kaplan-Meier curve.

Among patients who had received NST (either alone or followed by AST), 16.8% (19/113) experienced an event. The median time to recurrence, progression, or death was NR, and the estimated 5-year event-free rate was 78.0% (95% CI: 66.6–86.0%). Within this group, event rates differed significantly by pCR status (*p* = 0.0083, Figure 3). Specifically, among patients who achieved pCR, 8.2% (5/61) experienced recurrence or death; the median time to event was NR, and the 5-year event-free rate was 89.7% (95% CI: 76.3–95.7%). Among those with residual disease, 26.9% (14/52) experienced an event; the median time to event was also NR, and the 5-year event-free rate was 64.8% (95% CI: 45.6–78.7%).

Among patients who had not received NST (i.e., those treated with AST only or surgery only), 24.8% (29/117) experienced recurrence, disease progression, or death. The median time to event was not NR, and the 5-year event-free rate was 72.0% (95% CI: 61.6–80.0%).

## 4. Discussion

This observational, retrospective study provides significant insights into the treatment practices in Greece, focusing on the utilization of NST for women diagnosed with early-stage or locally advanced (stages II–III) TNBC during the period 2016–2022, preceding the approval of the first immunotherapy-based regimen in the neoadjuvant setting and exploring distinctive trends and temporal changes in the therapeutic approaches adopted in routine clinical practice.

NST, either alone or followed by AST, was utilized in 49.1% (95% CI: 42.5–55.8%) of patients, indicating moderate use. This rate aligns with the 54.5% recently reported by Ghafouri et al. (2022) [18] among patients diagnosed with stage I–III TNBC between 2013 and 2018 in the US. In more detail, 30.4% of patients in our cohort received NST only, and 18.7% further received AST after surgery (post-NST setting), while 43.9% underwent upfront surgery followed by AST (AST only), illustrating a similar pattern to that observed by Ghafouri et al. (37.1% NST only, 17.4% AST post-NST, and 45.5% AST only). Higher rates of NST use of approximately 68.5% were consistently reported by another US study among patients diagnosed with stage II–IIIB TNBC between 2008 and 2016 and by a Polish study among patients with I–IIIB treated between 2015 and 2020. In the latter study, 52.6% of patients were treated with NST only, while 15.8% received AST in the post-NST setting, and 18.4% AST only [19,22]. An even higher NST utilization rate of 85.0% was reported by a Dutch study focusing on stage III TNBC patients undergoing surgery between 2011 and 2015, although details for subsequent systemic therapy were not recorded [26]. In contrast, a study in Japan among patients with stage I–IIIB diagnosed between 2008 and 2015 found a significantly lower NST rate of 10.8% (NST only: 1.5%; NST followed by AST: 9.4%), and a 20.4% rate of AST-only in the face of a markedly high rate of surgery-only (68.8%). This possibly reflects the high prevalence of stage I (47.9%) among their patient population, which would typically necessitate less aggressive treatment. Further disaggregating the Japanese data, the NST rate increases to 18.3% for patients with stages II and III TNBC, though the rate of surgery-only remains high at 57.9%, indicating potential underutilization of NST in local practice [17,19]. Similarly, a study in Brazil showed that the vast majority of patients with early-stage or locally advanced TNBC diagnosed between 2012 and 2017 received AST only (75.3%), with only 24.6% receiving NST (NST only: 7.5%; NST followed by AST: 17.1%), further highlighting the potential variability in treatment practices across different regions and countries worldwide [23]. Although not directly comparable, variations in the treatment approaches may reflect differing demographic and clinical profiles of patients. It is important to note that we lack detailed insights into how individual healthcare providers account for these diverse characteristics in their treatment decisions, underscoring the complexity of clinical decision-making in the management of TNBC.

At a national level, our study revealed substantial variations in NST utilization across sites, with rates ranging from 0% to 93.8%. This variability suggests that treatment decisions may be shaped by institutional factors and potentially by patient-specific characteristics. Notably, *BRCA1/2* testing at diagnosis emerged as an important clinical parameter, reinforcing its critical role in guiding treatment decisions. It is worth highlighting that TNBC patients harboring *BRCA1/2* mutations tend to respond favorably to NST, achieving higher pCR rates than non-carriers following platinum-based NST or benefit from anthracycline- and taxane-based chemotherapy, and are prone to better overall survival and safer BCS [22,27,28]. Notably, in our study, 32.7% of those tested were positive for either *BRCA1* or *BRCA2* mutations; however, less than half of the cohort (43.9%) underwent testing, indicating the potential need for broader *BRCA* screening to fully assess mutation prevalence and drive informed treatment decisions. While larger tumor size was also identified as a significant predictor of NST use, its influence was minimal. Although larger tumors often guide physicians’ decisions to administer NST to reduce tumor burden preoperatively—potentially facilitating BCS and improving survival outcomes [26], our findings indicate that tumor size alone was not a major determinant of NST use. Given that, according to current clinical practice guidelines, all stage II–III TNBC patients are considered eligible for NST unless contraindicated [7,8,11], our findings suggest that there is room for improvement in neoadjuvant treatment adoption across breast cancer care institutions, calling for stronger multidisciplinary collaboration and commitment to standardized, guideline-driven strategies to ensure that all patients benefit from NST. The recent approval of newer targeted therapies, such as pembrolizumab—the first immunotherapy approved in the neoadjuvant setting—further reinforces the importance of consistent NST adoption to maximize therapeutic opportunities that might otherwise be missed postoperatively. Thus, coordinated efforts to align clinical practice with evidence-based treatment guidelines across all healthcare settings are essential to promote equitable access to optimal care, ultimately improving outcomes for stage II–III TNBC patients.

Interestingly, an increasing trend in the use of NST was observed, reaching a peak of 64% in the 2020–2022 period, coinciding with the most recent therapeutic advancements in this setting [14,15]. This trend mirrors global observations, such as in a Dutch study reporting that NST use among patients with non-metastatic (cT1-4N0-3M0) invasive BC doubled from 9% to 18% among all patients treated between 2011 and 2016, with the increase occurring in all clinical tumor categories except for cT4 (remaining stable at about 63.0% over the years) [20]. Notably, although a 24.2% rate of NST utilization among the subgroup of patients with TNBC was reported, specific temporal data were not available [20]. In another similar Dutch study analyzing NST utilization trends over a 10-year period, it was shown that both young and older BC patients were increasingly treated with NST followed by locoregional treatment. Notably, the most pronounced increase in the use of NST was observed in young patients with TNBC, rising from 44.3% in 2012–2015 to 82.3% in 2016–2020 [24].

Mastectomy, mainly unilateral, remained the predominant surgical option for treating TNBC regardless of the use of NST. However, the proportion of patients undergoing BCS seems to have gradually, though slightly, increased over the years, a shift that coincides with the increasing use of NST. Moreover, as expected, radiation therapy was also frequently used in the adjuvant setting to support surgical outcomes. As previously mentioned, by downstaging tumors before surgery, NST often makes previously inoperable tumors eligible for less extensive surgery, thereby preserving more breast tissue and improving cosmetic outcomes. If NST adoption continues to rise and more effective systemic treatments are incorporated into the therapeutic algorithm, NST’s effectiveness in reducing tumor size and eliminating cancer cells in regional lymph nodes will likely enhance the feasibility of BCS, making the clinical benefits of downstaging tumors even more evident [29]. Nevertheless, despite international guidelines routinely recommending BCS for early-stage TNBC [7,8], rates vary widely worldwide, and BCS remains largely underutilized [30,31]. Surgical decision-making after NST is influenced by multiple factors, including tumor characteristics, *BRCA* status, radiological response, and resource availability (e.g., access to radiotherapy, plastic surgery). Patient preferences as well as surgeon perspectives are also critical. For instance, results from the BrighTNess trial report that over half of stage II–III TNBC patients initially requiring mastectomy became eligible for BCS after NST (with eligibility rates rising from 76.5% to 83.8%), but only 56.0% of those women underwent BCS, and only 70% of those initially eligible ultimately underwent this surgical procedure [31]. Thus, while NST increases BCS eligibility among women with TNBC, many still opt for mastectomy, emphasizing the need for multidisciplinary team discussions, comprehensive patient counseling, and shared decision-making to optimize BCS utilization post-NST and enhance personalized care [30].

Regarding systemic therapy in the neoadjuvant setting, most patients received a regimen combining an anthracycline, cyclophosphamide, a platinum compound, and a taxane, predominantly featuring epirubicin, cyclophosphamide, paclitaxel, and carboplatin. The second most common combination comprised anthracyclines, cyclophosphamide, and taxanes. Such combinations were also commonly prescribed in the adjuvant setting, with epirubicin, cyclophosphamide, and docetaxel consistently being the predominant regimen in both settings. In the adjuvant setting, a similar frequency was also observed in the use of pyrimidine analogues, namely capecitabine, which was specifically administered to 88.7% of patients in the post-NST setting. Generally, regimens recorded in our study are those widely reported for the early-stage TNBC by various real-world studies, in line with the treatment guidelines [7,8,17,19,22]. Notably, the relatively high use of platinum-based regimens in the neoadjuvant setting observed in our study may reflect current guidelines recommending their consideration in selected patients with early-stage TNBC. These recommendations are based on emerging evidence showing that adding platinum compounds such as carboplatin to the standard neoadjuvant regimens could increase pCR rates [7,8]. However, the use of these agents has long been subject to debate, and the lack of conclusive data demonstrating their effects on long-term outcomes has prevented them from becoming a standard of care worldwide [32]. Indeed, earlier real-world studies consistently reported only limited usage [17,19,23]. Nevertheless, in line with our results, a significant increase in the incorporation of carboplatin in neoadjuvant regimens, reaching 57% in 2020, was also observed in a recent Polish study [22].

pCR achievement is undoubtedly the essential goal of neoadjuvant therapy for BC, directly affecting survival outcomes [33]. Following NST, more than half of our patients achieved pCR by the time of definitive surgery, and this was significantly associated with *BRCA1/BRCA2* positivity. Notably, testing positive for *BRCA1*, as well as for *BRCA1* and/or *BRCA2*, was strongly associated with higher odds of achieving pCR. This aligns with previous studies suggesting that *BRCA* mutations may confer increased sensitivity to chemotherapy, leading to higher pCR rates [28]. This response was more pronounced in patients treated with anthracyclines, cyclophosphamide, platinum compounds, and taxanes, achieving a pCR rate of 59.7%, compared to a pCR rate of 40.5% in patients treated with anthracyclines, cyclophosphamide, and taxanes without platinum compounds, highlighting the potential benefit of incorporating platinum compounds in NST regimens for TNBC. Notably, 41.0% of patients with pCR subsequently received adjuvant radiotherapy, while 39.3% did not receive any further treatment. Conversely, among those with residual disease at the time of definitive surgery, 54.2% went on to receive both AST and radiotherapy, and 31.3% received only adjuvant radiotherapy. This aligns with clinical practice, where patients who do not achieve a pCR often proceed to receive adjuvant treatment. Conversely, it is possible that patients who achieve a pCR in the neoadjuvant setting may be more likely to forego adjuvant treatment compared to those who do not achieve a pCR [19].

### Limitations and Future Directions

Potential limitations of this study are those inherent to its observational, retrospective design, including selection bias and information bias. Selection bias at the patient level was mitigated using consecutive enrollment, and selection bias at the site level was mitigated by including reference centers for BC from diverse geographic regions, ensuring generalizability to the broader Greek population with stage II–III TNBC. Information bias due to missing data was addressed by encouraging investigators to thoroughly review patient records and enter all data using standard eCRFs with validation rules, providing them with adequate time to gather necessary information as per usual practice. Data completeness was also assessed during feasibility visits, and patients were recruited in reverse chronological order, starting with the most recently diagnosed, to minimize the potential of missing data.

Although utilization of NST varied widely across Greek centers (0–93.8%), the study did not include institutional or clinician-level variables such as infrastructure, tumor board practices, or patient education that could contribute to heterogeneity. Such variability may lead to institutional bias, particularly if centers with higher NST utilization. In addition, NST and AST groups were not matched, and treatment selection inherently favored NST for larger or more biologically aggressive tumors. Consequently, differences in pCR, recurrence, and survival outcomes may partly reflect underlying tumor biology and selection effects rather than a causal impact of treatment duration. Future studies should incorporate center-level data and qualitative assessment of barriers to NST adoption in centers with low use.

Future prospective trials are essential to confirm these real-world insights and update the TNBC treatment algorithms. Larger, prospective, and more detailed studies need to deep-dive into the determinants of NST adoption, allowing for more reliable interpretation of results. Studies should specifically investigate patient-level factors, including clinical complexity, socioeconomic status, health literacy, and patient preferences, to understand why certain individuals may not receive NST or deny treatment, which remains an underexplored aspect of TNBC care. Additionally, future integration of label-free bioelectronic sensing platforms, such as Metas-Chip, could enhance real-time pCR prediction in neoadjuvant TNBC therapy. Finally, rigorous real-world efficacy studies are required to evaluate long-term outcomes, safety, and response patterns in routine clinical practice beyond clinical trial populations.

## 5. Conclusions

The TRINITY study offers critical retrospective real-world evidence on the management of early-stage and locally advanced TNBC in Greece. NST was used in nearly half of all patients, with substantial variation across institutions and increasing adoption over time. Patients who received NST were younger, more frequently underwent *BRCA* testing, and had larger and higher-grade tumors. More than half of patients receiving NST achieved a pCR, with *BRCA1/2* mutation carriers showing the greatest benefit. These impressive pCR rates reaffirm NST as the preferred treatment for eligible patients.

Our findings highlight the importance of a strong multidisciplinary approach and strict adherence to guidelines to deliver consistent, high-quality care. As new targeted and immunotherapy options emerge, refining NST strategies, improving access, and integrating innovative treatments into routine practice will be key to better outcomes.

## Figures and Tables

**Table 1:** Baseline demographics and clinical characteristics, overall and by NST utilization (*N* = 230).

Characteristic	Class	Overall (*N* = 230)	Non-NST (*N* = 117)	NST (*N* = 113)
Age (years), median (range)		53.1 (23.9–84.1)	58.5 (31.9–84.1)	48.0 (23.9–79.7)
Menopausal status, *n* (%)	Pre	91 (39.6)	30 (25.6)	61 (54.0)
	Post	128 (55.7)	79 (67.5)	49 (43.4)
	Unknown	11 (4.8)	8 (6.8)	3 (2.7)
BMI, *n* (%)	≥30 kg/m^2^	54 (23.5%)	25 (21.4)	29 (25.7)
	<30 kg/m^2^	176 (76.5%)	92 (78.6)	84 (74.3)
ECOG PS, *n* (%)	0	204 (88.7)	99 (84.6)	105 (92.9)
	1/2	24 (10.4)	17 (14.5)	7 (6.2)
	Unknown	2 (0.9)	1 (0.9)	1 (0.9)
Family history of BC, *n* (%)	Yes	76 (33.0)	31 (26.5)	45 (39.8)
	No	129 (56.1)	67 (57.3)	62 (54.9)
	Unknown	25 (10.9)	19 (16.2)	6 (5.3)
Histologic grade, *n* (%)	GX	1 (0.4)	0 (0.0%)	1 (0.9%)
	G1	1 (0.4)	0 (0.0%)	1 (0.9%)
	G2	38 (16.5)	22 (18.8%)	16 (14.2%)
	G3	184 (80.0)	91 (77.8)	93 (82.3)
	Unknown	6 (2.6)	4 (3.4)	2 (1.8)
Tumor size (mm), median (range)	-	27.5 (2.0–130.0)	25.0 (3.0–130.0)	30.0 (2.0–105.0)
Lymph node involvement, *n* (%)	Yes	134 (58.3%)	65 (55.6%)	69 (61.1%)
	No	96 (41.7)	52 (44.4%)	44 (38.9%)
Stage, *n* (%)	II	155 (67.4)	75 (64.1)	80 (70.8)
	III	75 (32.6)	42 (35.9)	33 (29.2)
*BRCA1/2* testing, *n* (%)	Yes	101 (43.9)	29 (24.8)	72 (63.7)
	No	112 (48.7)	77 (65.8)	35 (31.0)
	Unknown	17 (7.4)	11 (9.4)	6 (5.3)
*BRCA1* status, *n* (%)	Positive	32 (31.7)	7 (24.1)	25 (34.7)
	Negative	67 (66.3)	21 (72.4)	46 (63.9)
	Unknown	2 (2.0)	1 (3.5)	1 (1.4)
*BRCA2* status, *n* (%)	Positive	6 (5.9)	1 (3.5)	5 (6.9)
	Negative	88 (87.1)	23 (79.3)	65 (90.2)
	Unknown	7 (6.9)	5 (12.2)	2 (2.8)
*BRCA1/2* status, *n* (%)	Positive	33 (32.7)	7 (24.1)	26 (36.1)
	Negative	65 (64.4)	20 (69.0)	45 (62.5)
	Unknown	3 (2.9)	2 (6.9)	1 (1.4)
Ki 67%, median (range)	-	60.0 (5.0–100.0)	50.0 (5.0–100.100)	70.0 (7.0–100.0)
Surgery, *n* (%)	BCS	76 (33.0)	37 (31.6)	39 (34.5)
	Mastectomy	154 (67.0)	80 (68.4)	74 (65.5)

BC, breast cancer; BCS, breast-conserving surgery; BMI, body mass index; ECOG PS, Eastern Cooperative Oncology Group performance status; NST, neoadjuvant systemic therapy; Pre, time before menopause; Post, time after menopause; Yes, Presence of the characteristic; No, Absence of the characteristic; Unknown, Information not available or not recorded.

**Table 2:** Systemic therapy patterns and regimens in early-stage or locally advanced TNBC (*N* = 230).

Treatment Modality Regimen	NST Only (*N* = 70)	NST + AST (*N* = 43)	AST Only (*N* = 83)	Overall
**NST, *n* (%)**				
**Anthracyclines & Cyclophosphamide & Platinum compounds & Taxanes**	**46 (65.7)**	**21 (48.8)**		**67 (59.3)**
Epirubicin & Cyclophosphamide & Paclitaxel & Carboplatin	28 (40.0)	11 (25.6)	-	39 (34.5)
Epirubicin & Cyclophosphamide & Docetaxel & Carboplatin	15 (21.4)	9 (20.9)	-	24 (21.2)
Epirubicin & Cyclophosphamide & Paclitaxel & Docetaxel & Carboplatin	2 (1.4)	0 (0.0)	-	2 (1.8)
Other regimens	1 (2.9)	1 (2.3)	-	2 (1.8)
**Anthracyclines & Cyclophosphamide & Taxanes**	**22 (31.4)**	**15 (34.9)**	**-**	**37 (32.7)**
Epirubicin & Cyclophosphamide & Docetaxel	10 (14.3)	9 (20.9)	-	19 (16.8)
Doxorubicin & Cyclophosphamide & Paclitaxel	6 (8.6)	3 (7.0)	-	9 (8.0)
Doxorubicin & Cyclophosphamide & Docetaxel	2 (2.9)	2 (4.7)	-	4 (3.5)
Epirubicin & Cyclophosphamide & Paclitaxel & Docetaxel	2 (2.9)	1 (2.3)	-	3 (2.7)
Epirubicin & Cyclophosphamide & Paclitaxel	2 (2.9)	0 (0.0)	-	2 (1.8)
**Platinum compounds & Taxanes**	**0 (0.0)**	**5 (11.6)**	-	**5 (4.4)**
Carboplatin & Docetaxel	0 (0.0)	2 (4.7)	-	2 (1.8)
Carboplatin & Paclitaxel	0 (0.0)	2 (4.7)	-	2 (1.8)
Carboplatin & nab-Paclitaxel	0 (0.0)	1 (2.3)	-	1 (0.9)
**Other regimens** ^1^	**2 (2.9)**	**2 (4.7)**	-	**4 (3.6%)**
**Overall**	**70 (100%)**	**43 (100%)**	-	**113 (100%)**
**AST, *n* (%)**				
**Anthracyclines & Cyclophosphamide & Taxanes**	**-**	**1 (2.3)**	**38 (45.8)**	**39 (31.0)**
Epirubicin & Cyclophosphamide & Docetaxel	-	-	27 (32.5)	27 (21.4)
Epirubicin & Cyclophosphamide & Paclitaxel	-	-	7 (8.4)	7 (5.6)
Doxorubicin & Cyclophosphamide & Paclitaxel	-	-	2 (2.4)	2 (1.6)
Epirubicin & Cyclophosphamide & Paclitaxel & Docetaxel	-	-	2 (2.4)	2 (1.6)
Paclitaxel & Cyclophosphamide & Doxorubicin	-	1 (2.3)	0 (0.0)	1 (0.8)
**Anthracyclines & Cyclophosphamide & Platinum compounds & Taxanes**	**-**	**-**	**16 (19.3)**	**16 (12.7)**
Epirubicin & Cyclophosphamide & Docetaxel & Carboplatin	-	-	11 (13.3)	11 (8.7)
Epirubicin & Cyclophosphamide & Paclitaxel & Carboplatin	-	-	5 (6.0)	5 (4.0)
**Taxanes & Platinum compounds**			**6 (7.2)**	**6 (4.8)**
Paclitaxel & Carboplatin	-	-	3 (3.6)	3 (2.4)
Docetaxel & Carboplatin	-	-	2 (2.4)	2 (1.6)
Nab-Paclitaxel & Carboplatin	-	-	1 (1.2)	1 (0.8)
**Anthracyclines & Cyclophosphamide**			5 (6.0)	**5 (4.0)**
Epirubicin & Cyclophosphamide	-	-	5 (6.0)	5 (4.0)
**Folic acid analogues & Cyclophosphamide & Pyrimidine** **analogues**	**-**	**-**	5 (6.0)	**5 (4.0)**
Cyclophosphamide & Methotrexate & Fluorouracil	-	-	5 (6.0)	5 (4.0)
**Taxanes**	-	1 (2.3)	4 (4.8)	**5 (4.0)**
Paclitaxel	-	1 (2.3)	2 (2.4)	3 (2.4)
Other	-	-	2 (2.4)	2 (1.6)
**Anthracyclines and related substances & Cyclophosphamide & Pyrimidine analogues & Taxanes & Platinum compounds**	**-**	**-**	**3 (3.6)**	**3 (2.4)**
Epirubicin & Cyclophosphamide & Paclitaxel & Carboplatin & Fluorouracil	-	-	2 (2.4)	2 (1.6)
Epirubicin & Cyclophosphamide & Paclitaxel & Carboplatin & Capecitabine	-	-	1 (1.2)	1 (0.8)
**Cyclophosphamide & taxanes**	**-**	**-**	**3 (3.6)**	**3 (2.4)**
Cyclophosphamide & Paclitaxel & Docetaxel	-	-	2 (2.4)	2 (1.6)
Cyclophosphamide & Docetaxel	-	-	1 (1.2)	1 (0.8)
**Pyrimidine analogues**	**-**	38 (88.4)	1 (1.2)	**39 (31.0)**
Capecitabine	-	38 (88.4)	1 (1.2)	39 (31.0)
**Other regimens** ^1^	**-**	**3 (7.0)**	**2 (2.4)**	**5 (4.0)**
**Overall**	**-**	**43 (100%)**	**83** ^2^ **(100%)**	**126 (100%)**

^1^ Other includes regimens reported only once. ^2^ Treatment data for 18 patients receiving AST were not available. AST, adjuvant systemic therapy; NST, neoadjuvant systemic therapy; TNBC, triple-negative breast cancer.

## Data Availability

The original contributions presented in the study are included in the article/Supplementary Material; further inquiries can be directed to the corresponding author.

## Supplementary Materials

The following supporting information can be downloaded at: https://www.mdpi.com/article/10.3390/cancers17244023/s1, Table S1: Association of demographic and clinical characteristics with NST utilization; univariable logistic regression. Table S2: Association of baseline demographic and clinical characteristics with the physician’s decision to administer NST; multivariable logistic regression. Table S3: Baseline demographics and clinical characteristics among NST-treated patients, overall and by pCR (*N* = 113). Table S4: Association of baseline demographic and clinical characteristics with pCR among patients who received NST; univariable logistic regression.

## Abbreviations

AST	adjuvant systemic therapy
ASCO	American Society of Clinical Oncology
BC	breast cancer
BCS	breast-conserving surgery
BMI	body mass index
ECOG PS	Eastern Cooperative Oncology Group performance status
eCRF	electronic case report forms
ER	estrogen receptor
HER2	human epidermal growth factor receptor 2
ICF	informed consent obtainment
NST	neoadjuvant systemic therapy
NCCN	National Comprehensive Cancer Network
PR	progesterone receptor
pCR	pathological complete response
TNBC	triple-negative breast cancer
