# Supplementary material for: Real-World Neoadjuvant Systemic Therapy Utilization and Treatment Patterns in Patients with Early-Stage or Locally Advanced Triple-Negative Breast Cancer in Greece—The TRINITY Study
Source: Cancers (Basel). 2025 Dec 17;17(24):4023. doi: 10.3390/cancers17244023 (PMC12731357; doi:10.3390/cancers17244023)
Supplement: Supplementary file 1 [file cancers-17-04023-s001.zip › cancers-3977057-supplementary.pdf]

Table S1. Association of demographic and clinical characteristics with NST utilization; univariable logistic regression.

				95% CI		
**Parameter**	**Category vs. reference**	**n**	**OR**	**Lower limit**	**Upper limit**	***p*-value**
Age (years)	-	230	0.952	0.932	0.972	**< 0.0001**
Menopausal status	Pre- vs Post-menopausal	219	3.278	1.865	5.761	**< 0.0001**
BMI	≥ 30 kg/m^2 vs <30 kg/m^2	230	1.270	0.689	2.340	0.4432
ECOG PS	1/2 vs 0	228	0.388	0.154	0.976	**0.0443**
Family history of BC	Yes vs No	205	1.569	0.884	2.783	0.1237
Histologic grade	GX/G1/G2 vs G3	224	0.801	0.403	1.591	0.5256
Tumor size (mm)	-	230	1.023	1.003	1.043	**0.0221**
Lymph node involvement	Yes vs No	230	1.255	0.742	2.121	0.3975
TNM Stage	II vs III	230	1.358	0.780	2.363	0.2796
BRCA1/2 testing	Yes vs No/Unknown	230	5.329	3.018	9.408	**< 0.0001**
BRCA1 status	Positive vs Negative/Unknown	230	4.464	1.845	10.803	**0.0009**
BRCA2 status	Positive vs Negative/Unknown	230	5.370	0.617	46.693	0.1277
BRCA1/BRCA2 status	Positive vs Negative/Unknown	230	4.696	1.947	11.330	**0.0006**
Type of center	Private vs Public	230	6.382	3.549	11.477	**< 0.0001**

The modeled probability was NST vs. non-NST. Statistical significance was set at $p < 0.1$. Note: Distributions of examined parameters can be found in Table 1/Figure 2.

BC, Breast cancer; BMI, body mass index; CI, confidence interval; ECOG PS, Eastern Cooperative Oncology Group performance status; n, number of observations with available data; NST, neoadjuvant systemic treatment; OR, odds ratio.

Table S2. Association of baseline demographic and clinical characteristics with the physician's decision to administer NST; multivariable logistic regression

			95% CI		
**Parameter**	**Category vs. reference**	**OR**	**Lower limit**	**Upper limit**	**p-value**
Number of observations used in model: N=230					
Age at initial diagnosis (in years)		0.98	0.96	1.01	0.128
Tumor size (mm)		1.03	1.01	1.06	0.008
BRCA Testing	Yes vs No/Unknown	3.28	1.62	6.64	<0.001
Type of center	Private vs. Public	5.43	2.85	10.36	<0.001

The modeled probability was NST yes vs. NST no.

BRCA, BReast CAncer gene; CI, confidence interval; N, number of patients included in the analysis; NST, Neoadjuvant Systemic Treatment; OR, odds ratio.

Table S3. Baseline demographics and clinical characteristics among NST-treated patients, overall and by pCR (N = 113).

Characteristic	Class	Overall (N = 113)	Non-pCR (N = 52)	pCR (N = 61)
Age (years), median (range)		48.0 (23.9-79.7)	50.2 (34.2-79.7)	47.1 (23.9-76.2)
Menopausal status, n (%)	Pre	61 (54.0)	25 (48.1)	36 (59.0)
	Post	49 (43.4)	27 (51.9)	22 (36.1)
	Unknown	3 (2.7)	0 (0.0)	3 (4.9)
BMI, n (%)	≥30 kg/m^2	29 (25.7)	15 (28.8)	14 (23.0)
	<30 kg/m^2	84 (74.3)	37 (71.2)	47 (77.0)
ECOG PS, n (%)	0	105 (92.9)	46 (88.5)	59 (96.7)
	1/2	7 (6.2)	5 (9.6)	2 (3.3)
	Unknown	1 (0.9)	1 (1.9)	0 (0.0)
Family history of BC, n (%)	Yes	45 (39.8)	19 (36.5)	26 (42.6)
	No	62 (54.9)	33 (63.5)	29 (47.5)
	Unknown	6 (5.3)	0 (0.0)	6 (9.8)
Histologic grade, n (%)	GX	1 (0.9)	0 (0.0)	1 (1.6)
	G1	1 (0.9)	1 (1.9)	0 (0.0)
	G2	16 (14.2)	7 (13.5)	9 (14.8)
	G3	93 (82.3)	44 (84.6)	49 (80.3)
	Unknown	2 (1.8)	0 (0.0)	2 (3.3)
Tumor size (mm), median (range)	-	30.0 (2.0-105.0)	29.5 (2.0-105.0)	30.0 (13.0-80.0)
Lymph node involvement, n (%)	Yes	69 (61.1)	31 (59.6)	38 (62.3)
	No	44 (38.9)	21 (40.4)	23 (37.7)
Stage, n (%)	II	80 (70.8)	36 (69.2)	44 (72.1)
	III	33 (29.2)	16 (30.8)	17 (27.9)
BRCA1/2 testing, n (%)	Yes	72 (63.7)	27 (51.9)	45 (73.8)
	No	35 (31)	22 (42.3)	13 (21.3)
	Unknown	6 (5.3)	3 (5.8)	3 (4.9)
BRCA1 status, n (%)	Positive	25 (34.7)	4 (14.8)	21 (46.7)
	Negative	46 (63.9)	23 (85.2)	23 (51.1)
	Unknown	1 (1.4)	0 (0.0)	1 (2.2)
BRCA2 status, n (%)	Positive	5 (6.9)	1 (3.7)	4 (8.9)
	Negative	65 (90.3)	26 (96.3)	39 (86.7)
	Unknown	2 (2.8)	0 (0.0)	2 (4.4)
BRCA1/2 status, n (%)	Positive	26 (36.1%)	4 (14.8%)	22 (48.9%)
	Negative	45 (62.5%)	23 (85.2%)	22 (48.9%)
	Unknown	1 (1.4%)	0 (0.0)	1 (2.2%)
Ki 67%, median (range)	-	70.0 (7.0-100.0)	67.5 (7.0-100.0)	70.0 (10.0-95.0)

BC, breast cancer; BMI, body mass index; ECOG PS, Eastern Cooperative Oncology Group performance status; NST, neoadjuvant systemic therapy.

Table S4. Association of baseline demographic and clinical characteristics with pCR among patients who received NST; univariable logistic regression.

Parameter	Category vs. reference	n	OR	95% CI Lower limit	95% CI Upper limit	*p*-value
Age (years)		113	0.976	0.946	1.006	0.1188
Menopausal status	Pre- vs post-menopausal	110	1.767	0.827	3.778	0.1418
BMI	≥ 30 kg/m² vs <30 kg/m²	113	0.735	0.315	1.713	0.4753
ECOG PS	1/2 vs 0	112	0.312	0.058	1.682	0.1753
Family history of BC	Yes vs No	107	1.557	0.718	3.376	0.2620
Histologic grade	GX/G1/G2 vs G3	111	1.122	0.407	3.096	0.8240
Tumor size (mm)		113	0.995	0.972	1.018	0.6543
Lymph node involvement	Yes vs No	113	1.119	0.524	2.389	0.7708
TNM stage	II vs III	113	1.150	0.510	2.592	0.7355
BRCA1/2 testing	Yes vs No/Unknown	113	2.604	1.184	5.728	**0.0173**
BRCA1 status	Positive vs Negative/Unknown	113	6.299	1.997	19.864	**0.0017**
BRCA2 status	Positive vs Negative/Unknown	113	3.579	0.387	33.071	0.2611
BRCA1/BRCA2 status	Positive vs Negative/Unknown	113	6.767	2.151	21.287	**0.0011**
Ki 67%		106	1.010	0.993	1.028	0.236

The modeled probability was pCR vs non-pCR. Statistical significance was set at $p < 0.05$.
Note: For pCR achievement, patients with unknown pCR status were included in the “non-pCR” category. Also, patients with “No/Unknown” BRCA1/2 testing were included in the “Negative/Unknown” category for BRCA1 & BRCA2 status. Distributions of examined parametes can be found in Table S3.
BC, Breast cancer; BMI, body mass index; CI, confidence interval; ECOG PS, Eastern Cooperative Oncology Group performance status; n, number of observations with available data; NST, neoadjuvant systemic treatment; OR, odds ratio.
